# Supplementary material for: Fine Identification and Classification of a Novel Beneficial Talaromyces Fungal Species from Masson Pine Rhizosphere Soil
Source: J Fungi (Basel). 2022 Feb 3;8(2):155. doi: 10.3390/jof8020155 (PMC8877249; doi:10.3390/jof8020155)
Supplement: Supplementary file 1 [file jof-08-00155-s001.zip › jof-1582721-supplementary.pdf]

Supplementary materials

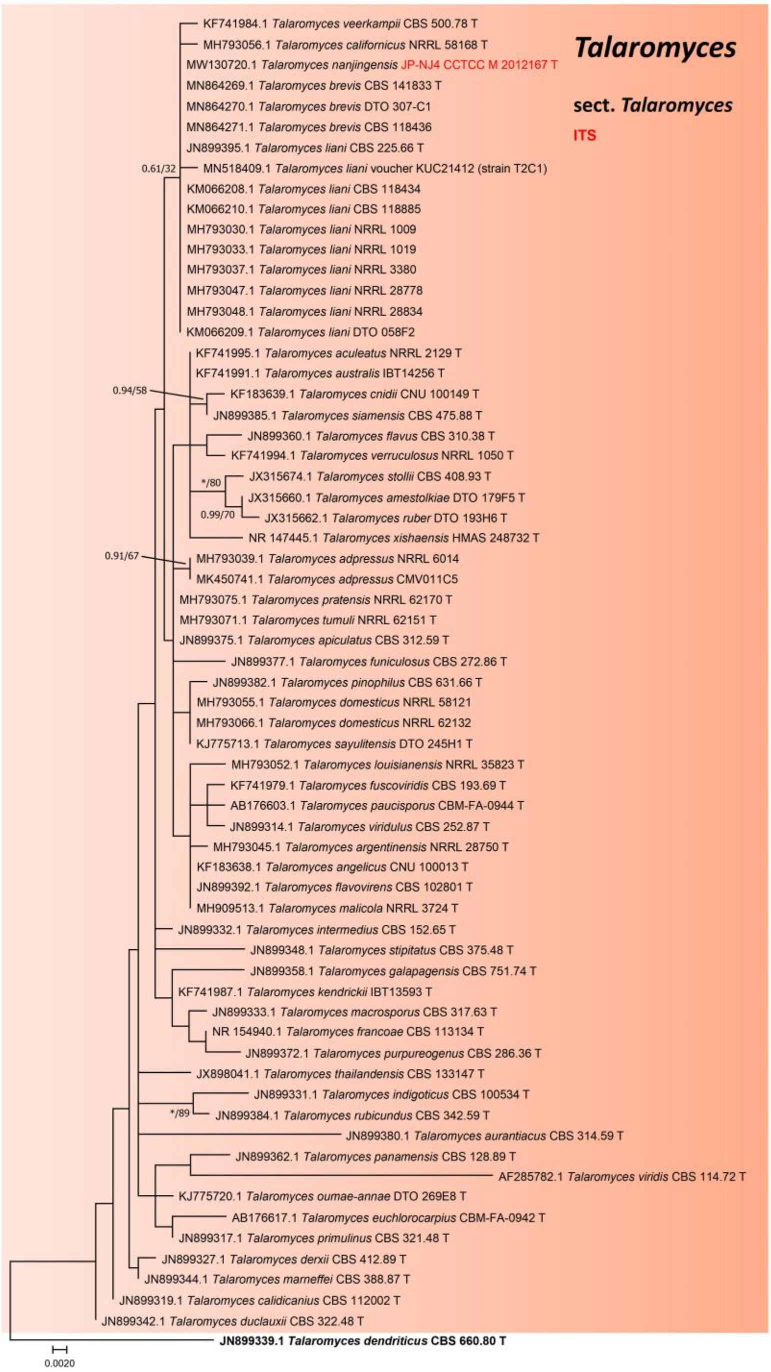

Figure S1 Maximum likelihood phylogeny of ITS regions for strain JP-NJ4 and other species classified in *Talaromyces* sect. *Talaromyces*. *Talaromyces dendriticus* (CBS_660.80_T) was chosen as out-group. Support in nodes is indicated above branches and is represented by posterior probabilities (BI analysis) and bootstrap values (ML analysis). Full support (1.00/100%) is indicated with an asterisk (*). Bootstrap values lower than 50 is hidden. Best-fit model of Bayesian Inference phylogeny according to BIC: GTR+F+I+G4; Best-fit model of Maximum likelihood phylogeny according to AIC: Tamura 3-parameter (T92) +G+I; alignment, ITS 467 bp. Scale bar: 0.0020 substitutions per nucleotide position. T indicates ex type. The strain with red font is the strain JP-NJ4 to be identified.

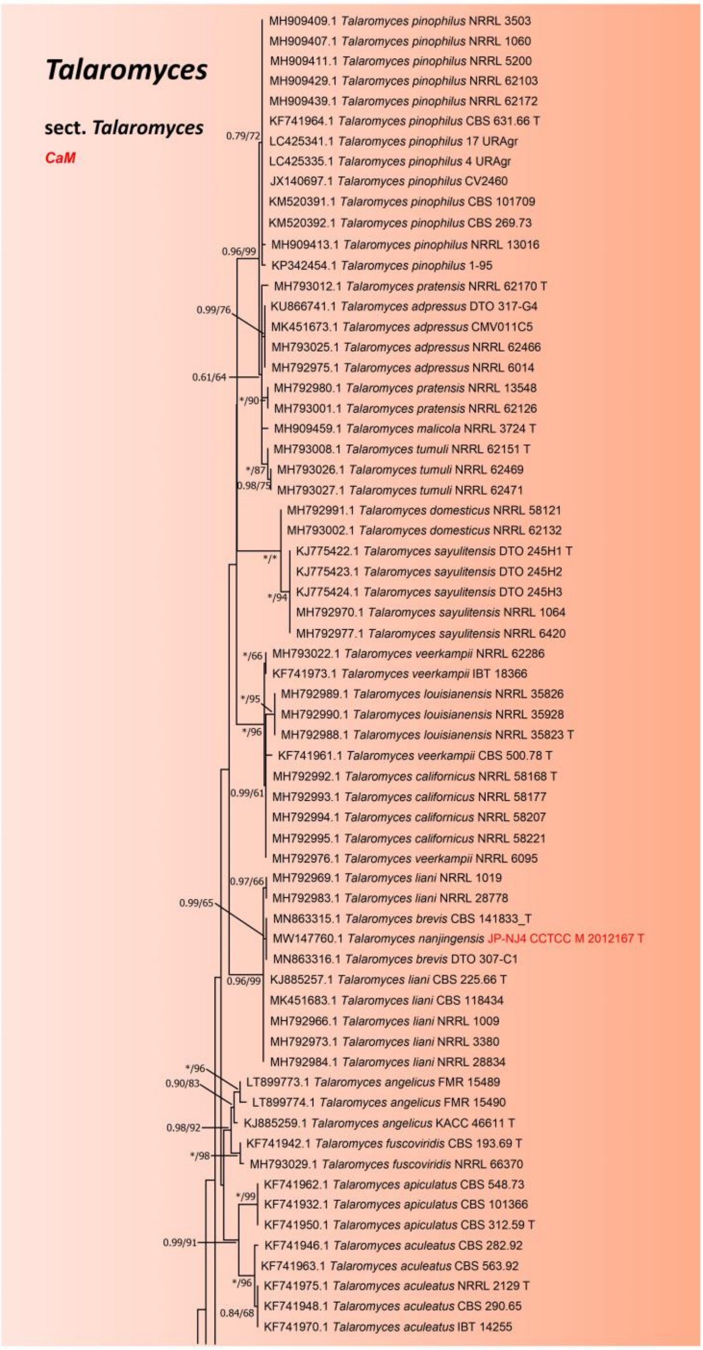

Talaromyces
sect. Talaromyces
CaM
MH909409.1 Talaromyces pinophilus NRRL 3503
MH909407.1 Talaromyces pinophilus NRRL 1060
MH909411.1 Talaromyces pinophilus NRRL 5200
MH909429.1 Talaromyces pinophilus NRRL 62103
MH909439.1 Talaromyces pinophilus NRRL 62172
KF741964.1 Talaromyces pinophilus CBS 631.66 T
LC425341.1 Talaromyces pinophilus 17 URAgr
LC425335.1 Talaromyces pinophilus 4 URAgr
JX140697.1 Talaromyces pinophilus CV2460
KM520391.1 Talaromyces pinophilus CBS 101709
KM520392.1 Talaromyces pinophilus CBS 269.73
MH909413.1 Talaromyces pinophilus NRRL 13016
KP342454.1 Talaromyces pinophilus 1-95
MH793012.1 Talaromyces pratensis NRRL 62170 T
KU866741.1 Talaromyces adpressus DTO 317-G4
MK451673.1 Talaromyces adpressus CMV011C5
MH793025.1 Talaromyces adpressus NRRL 62466
MH792975.1 Talaromyces adpressus NRRL 6014
MH792980.1 Talaromyces pratensis NRRL 13548
MH793001.1 Talaromyces pratensis NRRL 62126
MH909459.1 Talaromyces malicola NRRL 3724 T
MH793008.1 Talaromyces tumuli NRRL 62151 T
MH793026.1 Talaromyces tumuli NRRL 62469
MH793027.1 Talaromyces tumuli NRRL 62471
MH792991.1 Talaromyces domesticus NRRL 58121
MH793002.1 Talaromyces domesticus NRRL 62132
KJ775422.1 Talaromyces sayulitensis DTO 245H1 T
KJ775423.1 Talaromyces sayulitensis DTO 245H2
KJ775424.1 Talaromyces sayulitensis DTO 245H3
MH792970.1 Talaromyces sayulitensis NRRL 1064
MH792977.1 Talaromyces sayulitensis NRRL 6420
MH793022.1 Talaromyces veerkampii NRRL 62286
KF741973.1 Talaromyces veerkampii IBT 18366
MH792989.1 Talaromyces louisianensis NRRL 35826
MH792990.1 Talaromyces louisianensis NRRL 35928
MH792988.1 Talaromyces louisianensis NRRL 35823 T
KF741961.1 Talaromyces veerkampii CBS 500.78 T
MH792992.1 Talaromyces californicus NRRL 58168 T
MH792993.1 Talaromyces californicus NRRL 58177
MH792994.1 Talaromyces californicus NRRL 58207
MH792995.1 Talaromyces californicus NRRL 58221
MH792976.1 Talaromyces veerkampii NRRL 6095
MH792969.1 Talaromyces liani NRRL 1019
MH792983.1 Talaromyces liani NRRL 28778
MN863315.1 Talaromyces brevis CBS 141833_T
MW147760.1 Talaromyces nanjingensis JP-NJ4 CCTCC M 2012167 T
MN863316.1 Talaromyces brevis DTO 307-C1
KJ885257.1 Talaromyces liani CBS 225.66 T
MK451683.1 Talaromyces liani CBS 118434
MH792966.1 Talaromyces liani NRRL 1009
MH792973.1 Talaromyces liani NRRL 3380
MH792984.1 Talaromyces liani NRRL 28834
LT899773.1 Talaromyces angelicus FMR 15489
LT899774.1 Talaromyces angelicus FMR 15490
KJ885259.1 Talaromyces angelicus KACC 46611 T
KF741942.1 Talaromyces fuscoviridis CBS 193.69 T
MH793029.1 Talaromyces fuscoviridis NRRL 66370
KF741962.1 Talaromyces apiculatus CBS 548.73
KF741932.1 Talaromyces apiculatus CBS 101366
KF741950.1 Talaromyces apiculatus CBS 312.59 T
KF741946.1 Talaromyces aculeatus CBS 282.92
KF741963.1 Talaromyces aculeatus CBS 563.92
KF741975.1 Talaromyces aculeatus NRRL 2129 T
KF741948.1 Talaromyces aculeatus CBS 290.65
KF741970.1 Talaromyces aculeatus IBT 14255

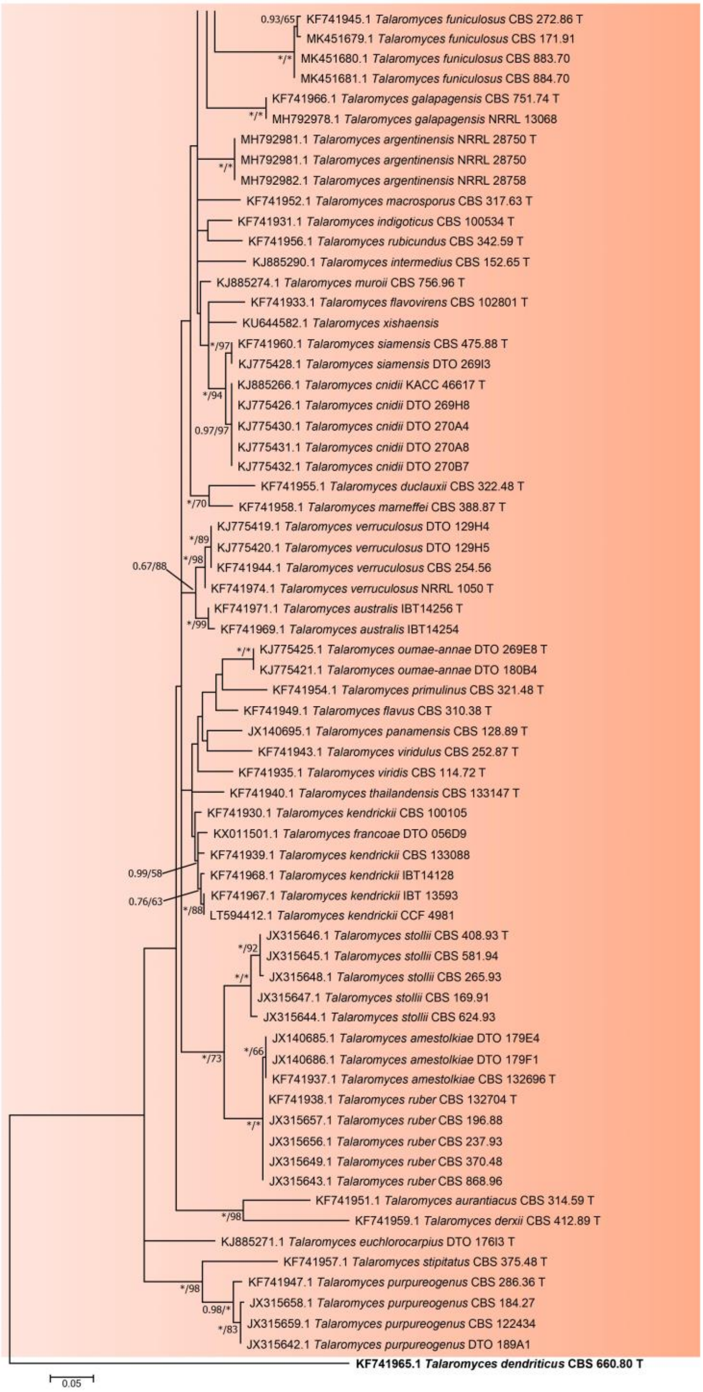

Figure S2 Maximum likelihood phylogeny of *CaM* gene regions for strain JP-NJ4 and other species classified in *Talaromyces* sect. *Talaromyces*. *Talaromyces dendriticus* (CBS_660.80_T) was chosen as out-group. Support in nodes is indicated above branches and is represented by posterior probabilities (BI analysis) and bootstrap values (ML analysis). Full support (1.00/100%) is indicated with an asterisk (*). Bootstrap values lower than 50

is hidden. Best-fit model of Bayesian Inference phylogeny according to BIC: SYM+I+G4; Best-fit model of Maximum likelihood phylogeny according to AIC: Kimura 2-parameter (K2) +G+I; alignment, *CaM* 475 bp. Scale bar: 0.05 substitutions per nucleotide position. T indicates ex type. The strain with red font is the strain JP-NJ4 to be identified.

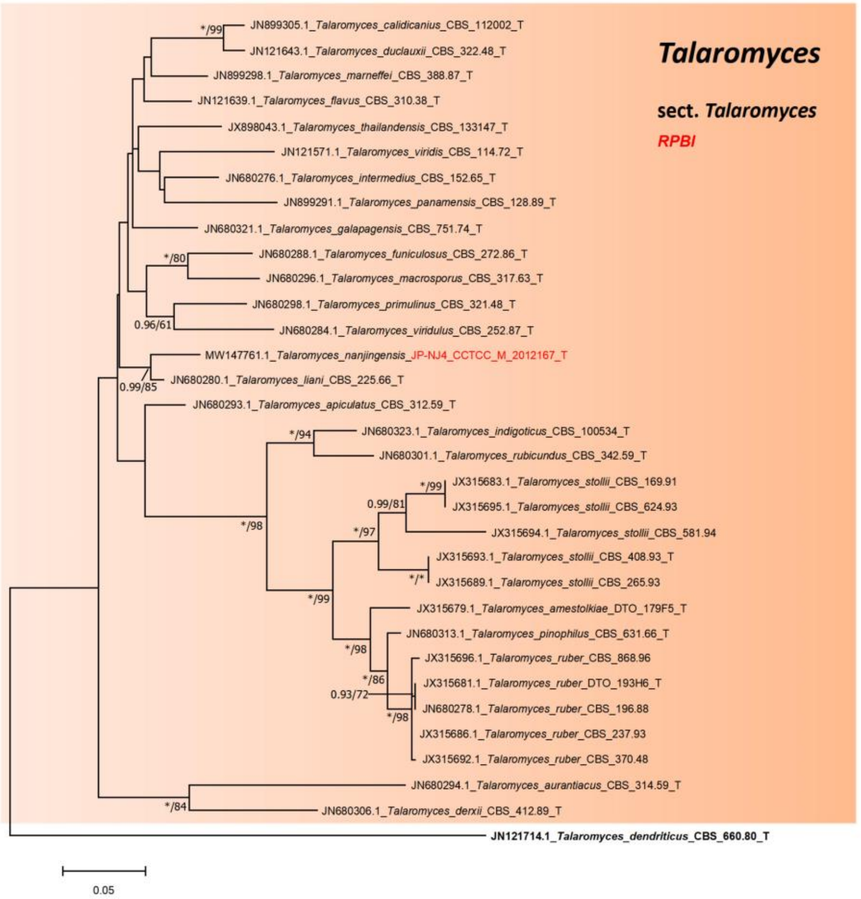

Figure S3 Maximum likelihood phylogeny of *RPBI* gene regions for strain JP-NJ4 and other species classified in *Talaromyces* sect. *Talaromyces*. *Talaromyces dendriticus* (CBS_660.80_T) was chosen as out-group. Support in nodes is indicated above branches and is represented by posterior probabilities (BI analysis) and bootstrap values (ML analysis). Full support (1.00/100%) is indicated with an asterisk (*). Bootstrap values lower than 50 is hidden. Support in nodes is indicated above branches and is represented by posterior probabilities (BI analysis) and bootstrap values (ML analysis). Full support (1.00/100%) is indicated with an asterisk (*). Best-fit model of Bayesian Inference phylogeny according to BIC: SYM+I+G; Best-fit model of Maximum likelihood phylogeny according to AIC: Kimura 2-parameter (K2) + G + I; alignment, *RPBI* 491 bp. Scale bar: 0.05 substitutions per nucleotide position. T indicates ex type. The strain with red font is the strain JP-NJ4 to be identified.

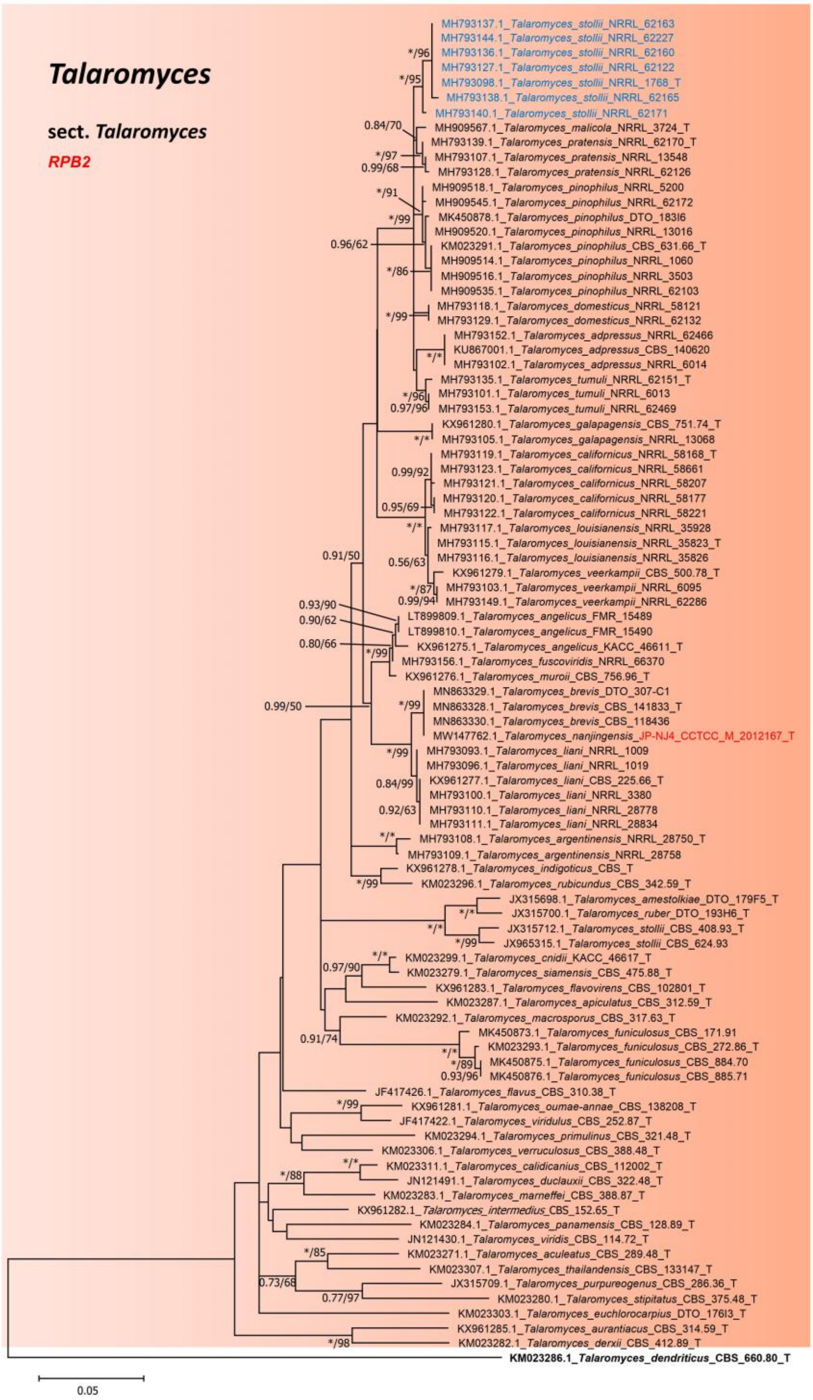

Figure S4 Maximum likelihood phylogeny of *RPB2* gene regions for strain JP-NJ4 and other species classified in *Talaromyces* sect. *Talaromyces*. *Talaromyces dendriticus* (CBS_660.80_T) was chosen as out-group. Support in nodes is indicated above branches and is represented by posterior probabilities (BI analysis) and bootstrap values (ML analysis). Full support (1.00/100%) is indicated with an asterisk (*). Bootstrap values lower

than 50 is hidden. Best-fit model of Bayesian Inference phylogeny according to BIC: K80 (K2P) +I+G4; Best-fit model of Maximum likelihood phylogeny according to AIC: Kimura 2-parameter (K2) +G+I; alignment, *RPB2* 718 bp. Scale bar: 0.05 substitutions per nucleotide position. T indicates ex type. The strain with red font is the strain JP-NJ4 to be identified.

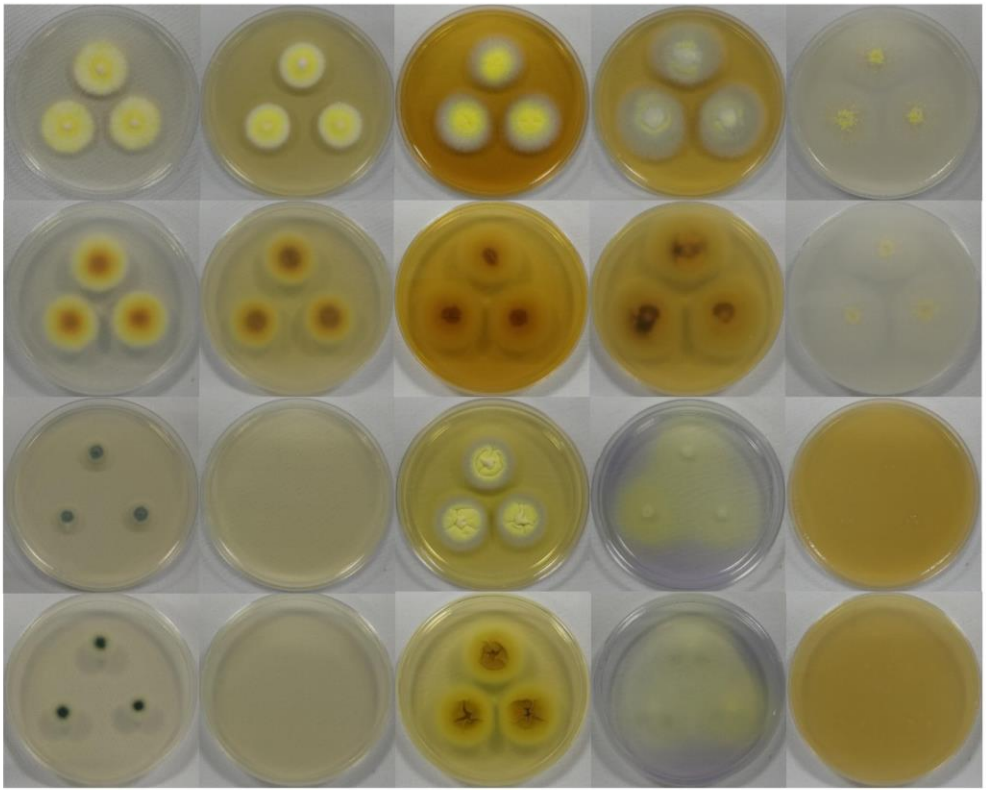

Figure S5 Macromorphological characters of strain JP-NJ4 (CCTCC M 2012167) (Inoculation at 25℃ for 7 days). Colonies from left to right: (the top two rows) CZ, CYA, MEA, MEAbl, OA and the reverse side corresponding to these media; (the bottom two rows) DG18, CYAS, YES, CREA, HAY and the reverse side corresponding to these media (The background color is white).

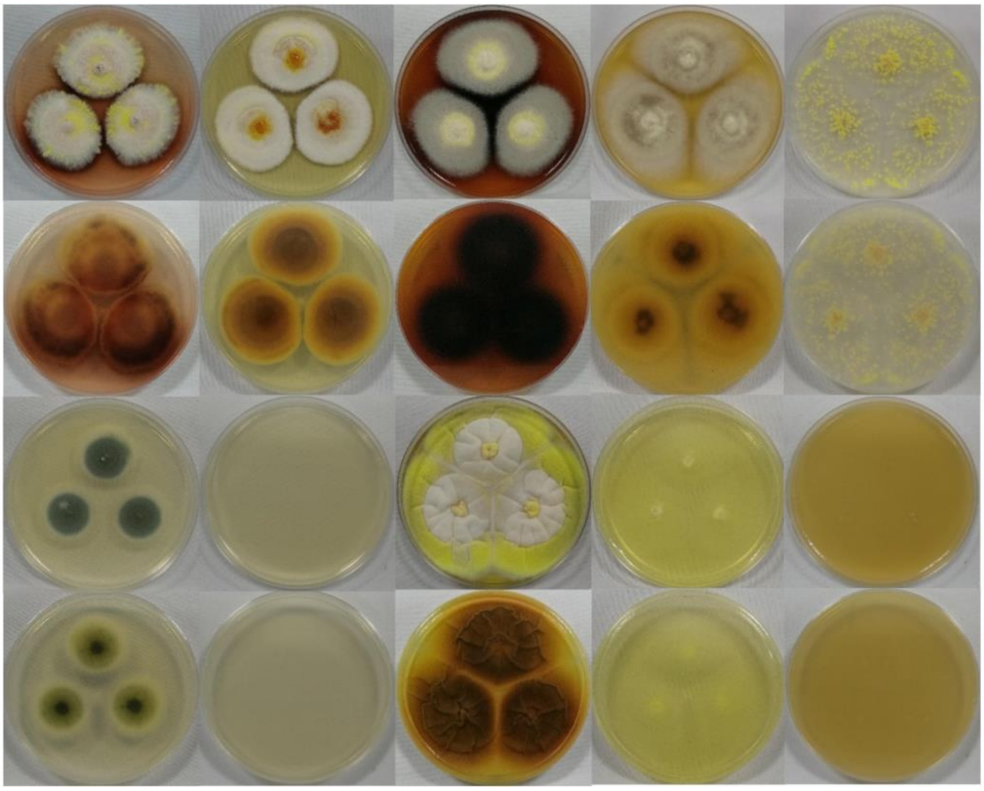

Figure S6 Macromorphological characters of strain JP-NJ4 (Inoculation at 25°C for 14 days). Colonies from left to right: (the top two rows) CZ, CYA, MEA, MEAbl, OA and the reverse side corresponding to these media; (the bottom two rows) DG18, CYAS, YES, CREA, HAY and the reverse side corresponding to these media. (The background color is white)

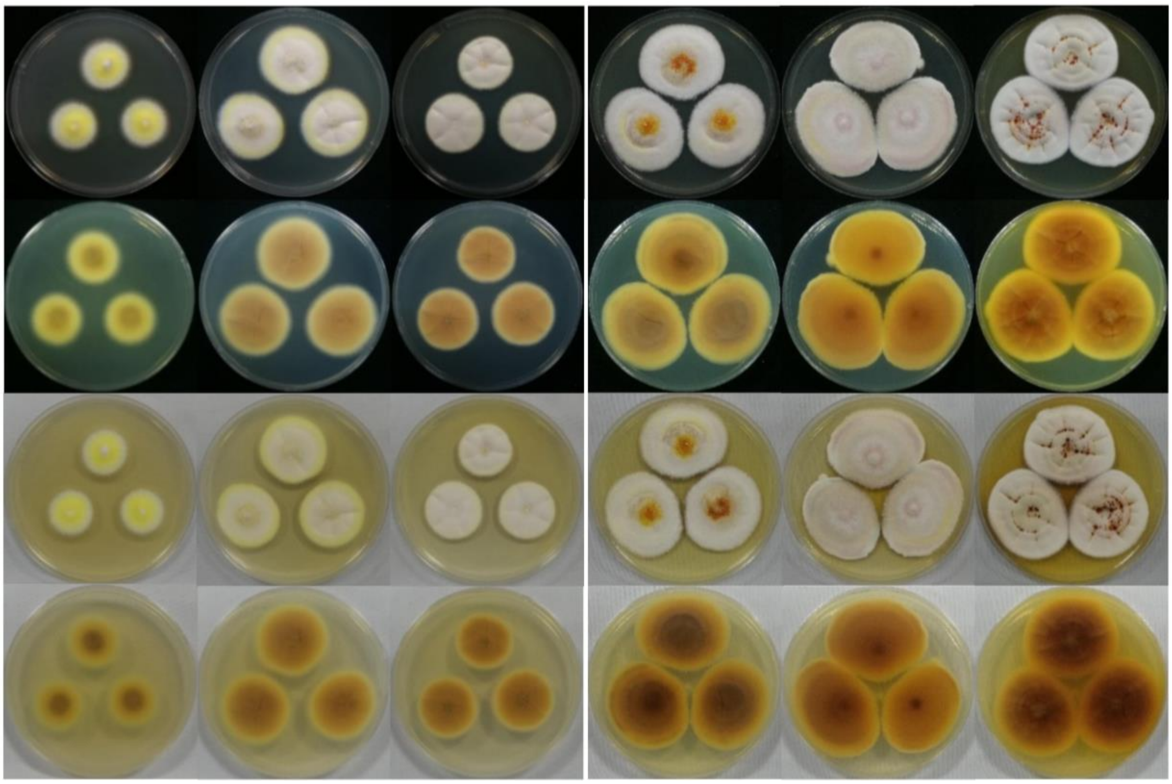

Figure S7 Macromorphology of strain JP-NJ4 under different temperature and culture days, Colonies from left to right: (the top two rows) CYA 25℃, 30℃, 37℃, inoculation for 7 days. CYA 25℃, 30℃, 37℃, inoculation for 14 days. and the reverse side corresponding to these media (The background color is black); (the bottom two rows) (The background color is white).

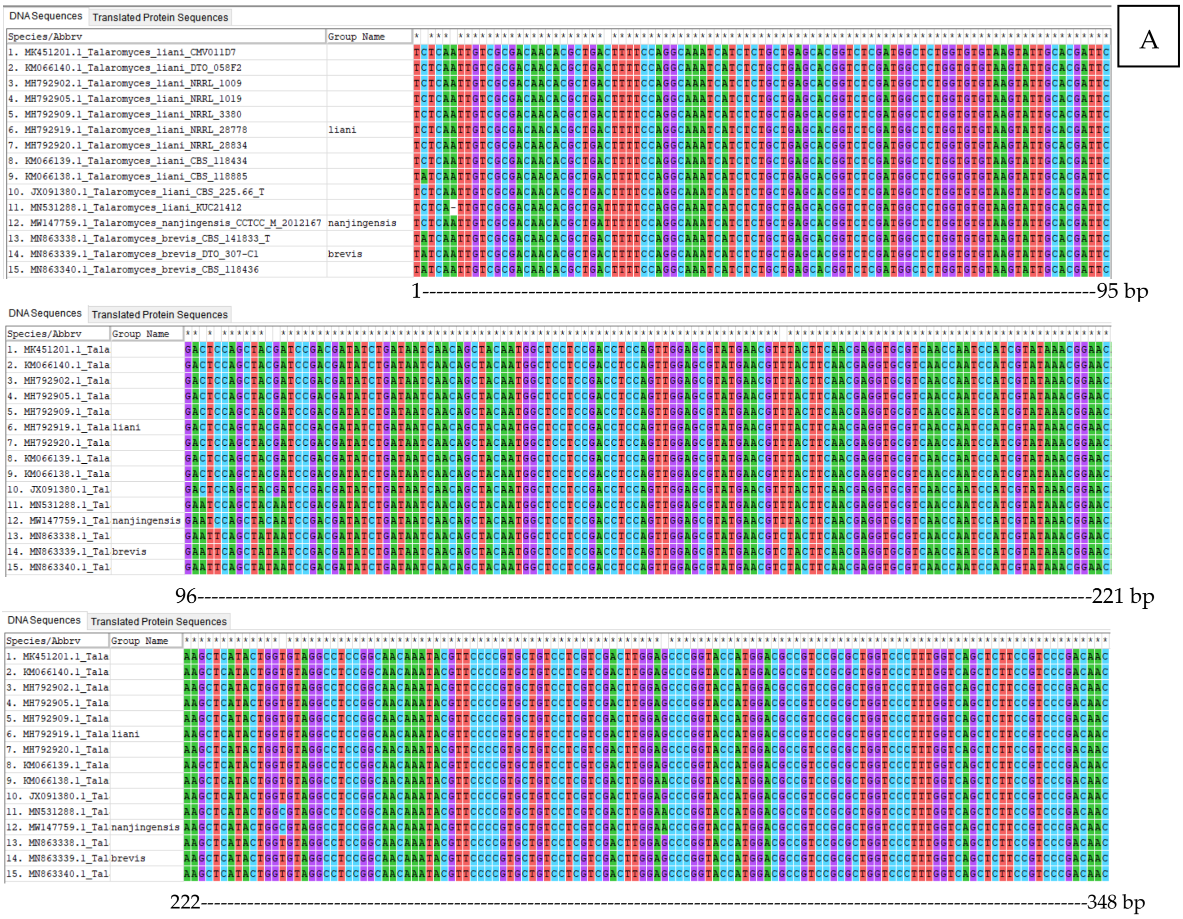
A
DNA Sequences
Translated Protein Sequences
Species/Abbrv
Group Name
1. MK451201.1_Talaromyces_liani_CMV011D7
2. KM066140.1_Talaromyces_liani_DTO_058F2
3. MH792902.1_Talaromyces_liani_NRRL_1009
4. MH792905.1_Talaromyces_liani_NRRL_1019
5. MH792909.1_Talaromyces_liani_NRRL_3380
6. MH792919.1_Talaromyces_liani_NRRL_28778
liani
7. MH792920.1_Talaromyces_liani_NRRL_28834
8. KM066139.1_Talaromyces_liani_CBS_118434
9. KM066138.1_Talaromyces_liani_CBS_118885
10. JX091380.1_Talaromyces_liani_CBS_225.66_T
11. MN531288.1_Talaromyces_liani_KUC21412
12. MW147759.1_Talaromyces_nanjingensis_CCTCC_M_2012167
nanjingensis
13. MN863338.1_Talaromyces_brevis_CBS_141833_T
14. MN863339.1_Talaromyces_brevis_DTO_307-C1
brevis
15. MN863340.1_Talaromyces_brevis_CBS_118436
1------95 bp
96------221 bp
222------348 bp

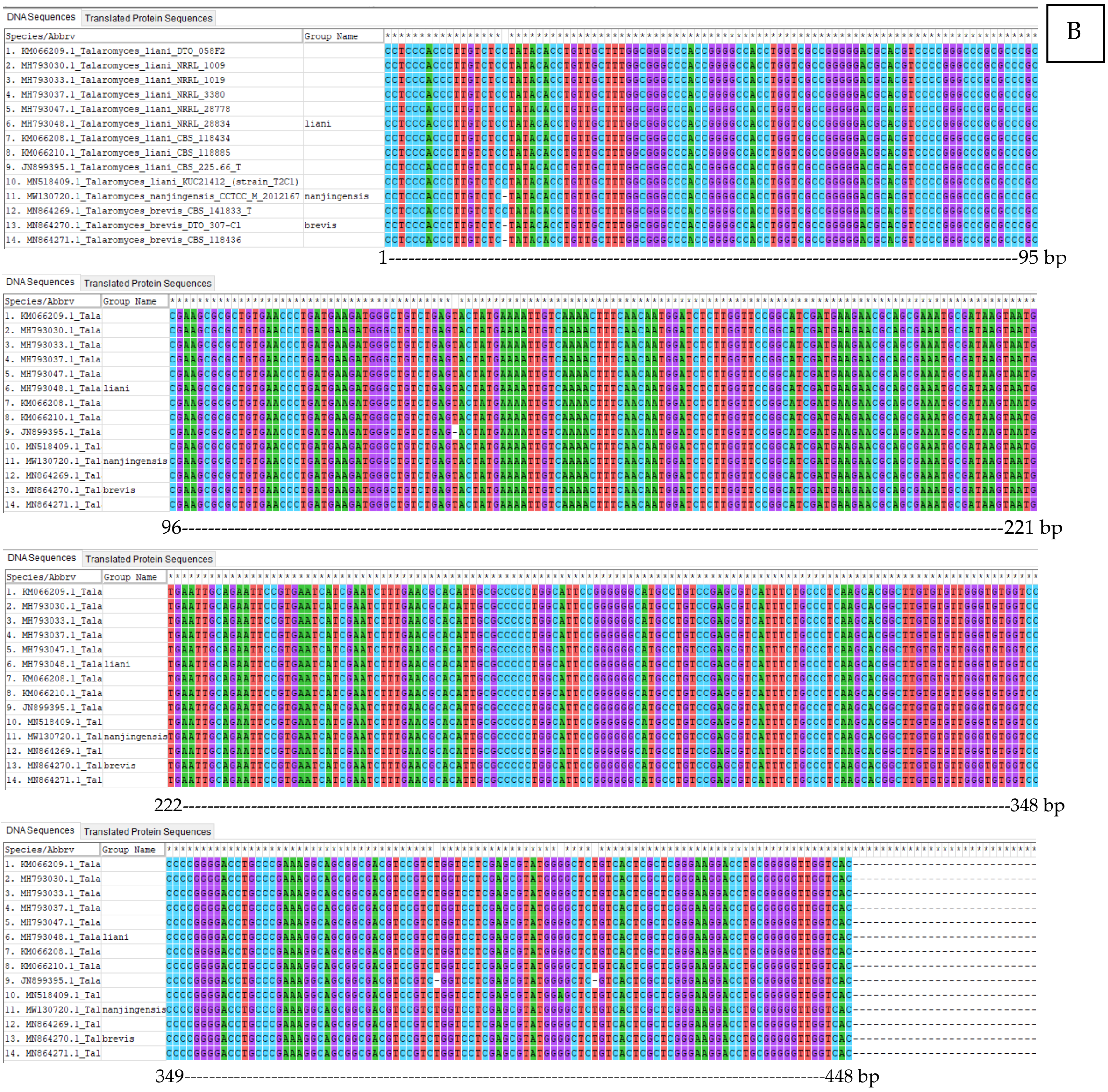

Figure S8 Base alignment and differences in specific genes of *Talaromyces nanjingensis*, *T. brevis* and *T. liani*. A. *BenA* gene, alignment, 348 bp; B. ITS region, alignment, 448 bp.

Table S1 Primers for amplification and sequencing of ITS and specific genes in strain JP-NJ4

Gene/Region	Primer name	Direction	Primer sequence (5′-3′)	Reference
Internal Transcribed Spacer rDNA area (ITS)	ITS1	Forward	TCCGTAGGTGAACCTGCGG	White et al. 1990
	ITS4	Reverse	TCCTCCGCTTATTGATATGC	
	V9G	Forward	TTACGTCCCTGCCCTTTGTA	de Hoog & van den Ende 1998
	LS266	Reverse	GCATTCCCAAACAACTCGACTC	Masclaux et al. 1995
β-tubulin (*BenA*)	Bt2a	Forward	GGTAACCAAATCGGTGCTGCTTTC	Lousie & Donaldson 1995
	Bt2b	Reverse	ACCCTCAGTGTAGTGACCCTTGGC	
Calmodulin (*CaM*)	CMD5	Forward	CCGAGTACAAGGAGGCCTTC	Rivera & Seifert 2011
	CMD6	Reverse	CCGATAGAGGTCATAACGTGG	
	CMD5′	Forward	CCGAGTACAAGGARGCCTTC	Hong et al. 2006
	CMD6′	Reverse	CCGATRGAGGTCATRACGTGG	
	CF1	Forward	GCCGACTCTTTGACYGAR	Peterson et al. 2005
	CF4	Reverse	CATCATRAGYTGGAC	
DNA-dependent RNA polymerase II (beta) largest subunit (*RPB1*)	F1843	Forward	ATTTYGAYGGTGAYGARATGAAC	Houbraken & Samson 2011 Primer R2623 is an internal primer for sequencing
	R3096	Reverse	GRACRGTDCCRTCATAYTTRACC	
	R2623	Internal	GCRTTGTTSARATCCTTMARRCTC	
DNA-dependent RNA polymerase II (beta) second largest subunit (*RPB2*)	5F	Forward	GAYGAYMGWGATCAYTTYGG	Liu et al. 1999 Used for identification of *Talaromyces*; Secondary primers for identification of *Penicillium*
	7CR	Reverse	CCCATRGCTTGYTTRCCCAT	
	5Feur	Forward	GAYGAYCGKGAYCAYTTCGG	Houbraken et al. 2012 Used for identification of *Penicillium*
	7CReur	Reverse	CCCATRGCYTGYTTRCCCAT	
	F311	Internal	CATGATYCARCGIAAYATGGA	Houbraken & Samson 2011
	R310	Internal	CCATRTTICGYTGRATCATGAA	

Table S2 Media required for the identification of strain JP-NJ4

Medium type and formulation		
**Czapek stock solution (CSS)**	**Trace elements stock solution (TESS)**	**Czapek's agar (CZ)**
$NaNO_3$ 30 g $MgSO_4{\cdot}7H_2O$ 5 g<br>KCl 5 g $FeSO_4{\cdot}7H_2O$ 0.1 g<br>dH_2O 100 ml	$CuSO_4{\cdot}5H_2O$ 0.5 g<br>$ZnSO_4{\cdot}7H_2O$ 0.1 g<br>dH_2O 100 ml	CSS 10 ml TESS 1 ml<br>Sucrose 30 g Agar 20 g<br>dH_2O 1000 ml
*Store at 4-10°C. (pitt 1979)	*Store at 4-10°C. (pitt 1979)	*Mix well and autoclave at 121°C for 15 min. (Raper and Thom 1949)
**Czapek Yeast Autolysate agar (CYA)**	**Czapek Yeast Autolysate agar with 5% NaCl (CYAS)**	**Blakeslee's Malt extract agar (MEAbl)**
CSS 10 ml TESS 1 ml<br>Sucrose 30 g Agar 20 g<br>K_2HPO_4 1 g dH_2O 1000 ml<br>Yeast extract (Difco) 5 g	CSS 10 ml TESS 1 ml<br>Sucrose 30 g Agar 20 g<br>K_2HPO_4 1 g dH_2O 1000 ml<br>NaCl 50 g<br>Yeast extract (Difco) 5 g	Glucose 20 g Agar 20 g<br>TESS 1 ml dH_2O 1000 ml<br>Malt extract (Oxoid) 20 g<br>Peptone (Oxoid) 1 g
*Mix well and autoclave at 121°C for 15 min. pH 6.2 ± 0.2. (Pitt 1979)	*Mix well and autoclave at 121°C for 15 min. pH 6.2 ± 0.2. (Visagie et al. 2014)	*Mix well and autoclave at 121°C for 15 min. pH 5.3 ± 0.2. (Blakeslee 1915)
**Malt Extract agar (MEA)**	**Dichloran 18% Glycerol agar (DG18)**	**Yeast extract sucrose agar (YES)**
TESS 1 ml Agar 20 g<br>dH_2O 1000 ml<br>Malt extract (Oxoid CM0059) 50 g	TESS 1 ml Agar 20 g<br>dH_2O 1000 ml<br>Chloramphenicol 0.05 g<br>Glycerol (anhydrous) 220 g<br>Dichloran-Glycerol-agar-base (Oxoid) 31.5 g	Sucrose 150 g TESS 1 ml<br>Agar 20 g dH_2O 885 ml<br>$MgSO_4{\cdot}7H_2O$ 0.5 g<br>Yeast extract (Difco) 20 g
*Mix well and autoclave at 115°C for 10 min. pH 5.4 ± 0.2. (Samson et al. 2010)	*Mix well and autoclave at 121°C for 15 min. After autoclaving, add 0.05 g chlortetracycline. pH 5.6 ± 0.2. (Hocking and Pitt 1980)	*Mix well and autoclave at 121°C for 15 min. p H 6.5 ± 0.2. (Frisvad 1981)
**Oatmeal agar (OA)**	**Creatine sucrose agar (CREA)**	**Hay infusion agar (HAY)**

TESS 1 ml Agar 20 g<br>Oatmeal /flakes 30 g dH_2O 1000 ml<br>1. First autoclave flakes in 1000 ml d H_2O. (121°C for 15 min)<br>2.Squeeze mixture through cheese cloth and use flow through, topping up to 1000 ml with d H_2O with 20 g agar	Sucrose 30 g KCl 0.5 g<br>Creatine·$1H_2O$ 3 g TESS 1 ml<br>Agar 20 g dH_2O 1000 ml<br>$K_3PO_4 \cdot 7H_2O$ 1.6 g<br>$MgSO_4 \cdot 7H_2O$ 0.5 g<br>$FeSO_4 \cdot 7H_2O$ 0.01 g<br>Bromocresol purple 0.05 g	Hay TESS 1 ml<br>dH_2O 1000 ml Agar 20 g<br>1. Fill a 1-liter beaker with hay<br>2. Add water and boil until the beaker becomes the color of weak tea.<br>3. Filter the water through cleesecloth or carefully pour off the water leaving the hay behind.
* Autoclave at 121°C for 15 min. pH 6.5 ± 0.2. (Samson et al. 2010)	*Mix well and autoclave at 121°C for 15 min. pH 8.0 ± 0.2. (Frisvad 1981)	*Mix well and autoclave at 121°C for 20 min. (David 1993)

Notes: CSS is the abbreviation of Czapek stock solution; TESS is the abbreviation of Trace elements stock solution
